# Efficacy of Online Intervention for ADHD: A Meta-Analysis and Systematic Review

**DOI:** 10.3389/fpsyg.2022.854810

**Published:** 2022-06-28

**Authors:** Songting Shou, Shengyao Xiu, Yuanliang Li, Ning Zhang, Jinglong Yu, Jie Ding, Junhong Wang

**Affiliations:** ^1^Department of Pediatrics, Dongzhimen Hospital, Beijing, China; ^2^Graduate School, Beijing University of Chinese Medicine, Beijing, China; ^3^Department of Integrative Oncology, China-Japan Friendship Hospital, Beijing, China

**Keywords:** online therapy, cognitive behavioral therapy, meta-analysis, systematic review, attention deficit/hyperactivity disorder

## Abstract

**Background:**

With the popularity of computers, the internet, and the global spread of COVID-19, more and more attention deficit hyperactivity disorder (ADHD) patients need timely interventions through the internet. At present, there are many online intervention schemes may help these patients. It is necessary to integrate data to analyze their effectiveness.

**Objectives:**

Our purpose is to integrate the ADHD online interventions trials, study its treatment effect and analyze its feasibility, and provide reference information for doctors in other institutions to formulate better treatment plans.

**Methods:**

We searched PubMed, EMBASE and Cochrane libraries. We didn't limit the start date and end date of search results. Our last search was on December 1, 2021. The keyword is ADHD online therapy. We used the Cochrane bias risk tool to assess the quality of included studies, used the standardized mean difference (SMD) as an effect scale indicator to measure data. Random effects model, subgroup analysis were used to analyze the data.

**Results:**

Six randomized controlled trials (RCTs) were identified, including 261 patients with ADHD. These studies showed that online interventions was more effective than waiting list in improving attention deficit and social function of adults and children with ADHD. The attention deficit scores of subjects were calculated in six studies. The sample size of the test group was 123, the sample size of the control group was 133, and the combined SMD was −0.73 (95% confidence interval: −1.01, −0.44). The social function scores of subjects were calculated in six studies. The sample size of the experimental group was 123 and the control group was 133. The combined SMD was −0.59 (95% confidence interval: −0.85, −0.33).

**Conclusions:**

The results show that online interventions of ADHD may be an effective intervention. In the future, we need more online intervention researches to improve the symptoms of different patients, especially for some patients who have difficulties in accepting face-to-face treatment.

## Introduction

Attention deficit hyperactivity disorder (ADHD) is currently considered to be a developmental disorder. It occurs in childhood and is characterized by difficulty in concentration, hyperactivity, and impulsive personality. The pathogenesis of ADHD has not been thoroughly studied, and various studies show the complexity of the disease (Faraone et al., 2015). This disease will not only affect an individual's learning function, but also bring emotional problems and daily life problems (Reimherr et al., 2020), such as marriage (Anastopoulos et al., 2009), and friendship (Pringsheim et al., 2015). At present, the prevalence of ADHD in children has exceeded 5% (Kuja-Halkola et al., 2021). At the same time, many children's academic performance is affected by the emergence of this disease (Matthys et al., 1999). Even adult college students will be troubled by ADHD, resulting in learning difficulties (Anastopoulos et al., 2016). Although many drugs can improve some symptoms of ADHD, it is still difficult to change their cognitive function and improve their academic performance. Because these drugs can only improve attention in a short time, they often do not help to learn the work that requires long-term attention, and even hinder the completion of related tasks (Bidwell et al., 2011). On the contrary, researchers found that nonpharmaceutical research can help patients effectively maintain the progress of various learning and life functions for a long time, and has the advantage of fewer side effects. Therefore, researchers gradually focus on the research of nonpharmaceutical treatment (Molina et al., 2009).

In addition, in some areas, the diagnosis, treatment and care of ADHD are often difficult to be continuous because of insufficient intervention intensity and medical support (Zima et al., 2010). To solve this problem, some scholars began to develop a remote intervention to help patients who have difficulties in communicating face-to-face with doctors in medical institutions for various reasons (Epstein et al., 2011). Some studies have shown that the remote intervention has a high completion rate, and both doctors and patients are willing to participate in remote intervention. It is believed that there are still some differences between teletherapy and other treatments (Vander Stoep and Myers, 2013). If professional training can be carried out, the curative effect of teletherapy may be improved (Vander Stoep and Myers, 2013). Because face-to-face communication based on network is more conducive to the communication between doctors and patients than telephone communication, some studies have begun to explore remote face-to-face intervention through network. A study showed that after network-based education and nursing education for ADHD families, the symptom reports of parents of ADHD children have been significantly improved, which has a certain effect of adjuvant treatment. Another study showed through a controlled experiment that after the network-based education and nursing education in addition to drug treatment for ADHD families, the attention report of ADHD children's parents has achieved a better curative effect than that of patients' families without remote intervention, and has a certain adjuvant treatment effect. These studies also believe that the functional impairment of ADHD patients may still need to be treated through behavioral training (Vander Stoep and Myers, 2013; Epstein et al., 2016). A survey shows that adult tic patients are often treated with drugs and rarely have the opportunity to receive psychotherapy (Kessler et al., 2006). These three studies reflect the importance of long-distance treatment therapy development for the treatment of ADHD from different aspects.

Nowadays, as one of the means of the remote treatment, online intervention can help some patients solve the problem that it is difficult to get treatment in time. Especially during the novel coronavirus epidemic, the reduction of personnel mobility can help to prevent the spread of the epidemic. But the efficacy of online interventions has not been comprehensively analyzed by researchers. Therefore, this study will analyze the effect of online treatment by analyzing the results of randomized controlled trials of online treatment of ADHD, so as to evaluate their efficacy in reducing cognitive and social disorders of this disease and provide decision-making basis for clinical workers.

## Methods

### Search Strategy

Our study was done in accordance with the guidance of the PRISMA statement (Page et al., 2021). We searched PubMed, EMBASE and Cochrane libraries. We didn't limit the start date and end date of search results. Our last search was on December 1, 2021. The keyword is ADHD treated online. Search terms included “online therapy” and “attention deficit hyperactivity disorder,” along with numerous other related terms. We limited the study language to English. The full search strategies are detailed in the additional files.

Three reviewers (SST, XXY, and LYL) working independently considered the potential eligibility of each of the abstracts generated by the search strategy. We will ask the authors of some studies for help by email so that we can obtain all the data. Articles are screened independently by the three reviewers and any differences will be settled through negotiation.

### Inclusion and Exclusion Criteria

Our study only included RCT, and the intervention form of the experimental group must be online intervention. The intervention form of the control group should be waiting list. The results were evaluated within 1 month after treatment. The subjects were patients with ADHD. The evaluation tool is the relevant scale used by ADHD. Considering that there are many scales to evaluate attention deficit and social function, we choose validated scales such as ADHD-Rating Scale (ADHD-RS) (Dupaul et al., 1998) and ADHD current symptoms scale (ADHD CSS) (Fuchs, 1999), ADHD self-report scale (ASRS) (Kessler et al., 2005), etc. The score change of ADHD related scale was used as the outcome index. We excluded studies in which the control group also used cognitive behavioral therapy or did not score the social function of patients with ADHD in the study.

### Study Selection and Data Extraction

This study extracted these data from the full text of various studies: (1) The name of the first author; (2) Year of publication of the study; (3) Sample size of the study; (4) The mean and standard deviation (SD) of the subject's age or the subject's age range; (5) Outcome indicators; (6) Research intervention methods; (7) Duration of intervention. The above information is summarized in Table 1, reviewed by three reviewers, and a unified opinion is formed after negotiating different opinions. The researchers checked repeatedly to ensure the accuracy of test data input.

**Table 1:** Characteristics of the literature included in the study.

**Researcher**	**Year**	**Nationality**	**Sample size of intervention group (Mean age or range)**	**Sample size of control group (Mean age or range)**	**Intervention methods**	**Duration of interventio**	**ADHD outcome measures**	**Social function outcome measures**	**JADAD**
Ashley F. McDermott	2016	USA	16 (8–12)	17 (8–12)	FFM/Waitlist	8 week	ADHD-RS	CGI	5
Birger Moell,	2014	Sweden	26 (36.3)	27 (37.3)	CBT/Waitlist	6 week	ASRS	SDS	6
George J. DuPaul	2017	USA	13 (4.52)	15 (4.27)	BPT/Waitlist	10 week	Conners (I/O)	P-DCI	5
Nike Franke	2016	Australia	27 (3–4)	26 (3–4)	TPOL/Waitlist	16 week	Con Hyp/Inatt	Con SocFunct	5
Penny Corkum	2015	Canada	28 (8.82)	30 (8.38)	OWL/Waitlist	6 week	Conners3-T ADHD Index T scores	Parent impairment ratings raw score	7
Richard Pettersson	2016	Sweden	13 (38.92)	18 (33.78)	iCBT/Waitlist	10 week	CSS	COPM performanc	5

*FFM, Feed-Forward Modeling System; CBT, Cognitive Behavioral Bherapy; BPT, Behavioral Parent Training; TPO, Triple P Online; OWL, Online Web Learning; iCBT, Internet-based Cognitive Behavioral Therapy; ADHD-RS, ADHD–Rating Scale; CGI, Clinical Global Impression; ASRS, ADHD Self-Report Scale; SDS, Sheehan Disability Scale; ADHD CSS, ADHD Current Symptoms Scale; Conners (I/O), Conners Inattention/Overactivity; P-DCI, parent–child dysfunctional interactions; COPM, Canadian Occupational Performance Measure; JADAD, Jadad scale is a tool to independently evaluate the quality of clinical trial methodology (1–3 points are regarded as low quality and 4–7 points are regarded as high quality)*.

### Effect Size

We used Review Manager 5.3 to analyze the data of each study. The efficacy was evaluated according to the mean and SD of each study (Weisman et al., 2013). By comparing the data of the experimental group and the control group, the overall differences between the two groups were compared. The total clinical score, social function score of the two groups were drawn by forest plot.

### Quality Assessments

In this study, the quality of the article was evaluated according to the relevant standards in the Cochrane bias risk assessment tool. Evaluations were made from selection (including random sequence and assignment concealment), implementation (including blind method of researchers and subjects), measurement (blind evaluation of research results), follow-up (integrity of results data), report (selective report of research results), and others (other sources of error). The judgment results of “low-risk bias,” “high-risk bias,” and “unclear” were made according to the bias risk assessment criteria. It is displayed in different colors (green, red, and yellow) (Supplementary Figures 1, 2).

### Statistical Analyses

In the study, we take the scale scores of healthy people without any functional impairment as the benchmark. The greater the difference between the scores in each group and this benchmark, the more serious the functional impairment. We will count this difference as the basis for analyzing various studies. When analyzing the data, we used Review Manager 5.3 for meta-analysis to quantitatively synthesize the data. Considering the different evaluation scales of each experiment, we select the random effect model and select standard mean difference (SMD) as the effect scale index of the measurement data (Higgins and Green, 2011), expressed as 95% confidence interval (CI). According to the *Z* or *U* value or chi-square value, the probability *p*-value under this statistic is obtained, and the significance level is set to 0.05. If *P* < 0.05, the pooled effect of these studies is statistically significant. An I-squared value of 25% is considered as low, 50% as moderate, and 75% as high heterogeneity (Higgins et al., 2003). We analyze whether the six included studies have publication bias through Stata 14.

## Results

### Included Studies

**Figure 1:**
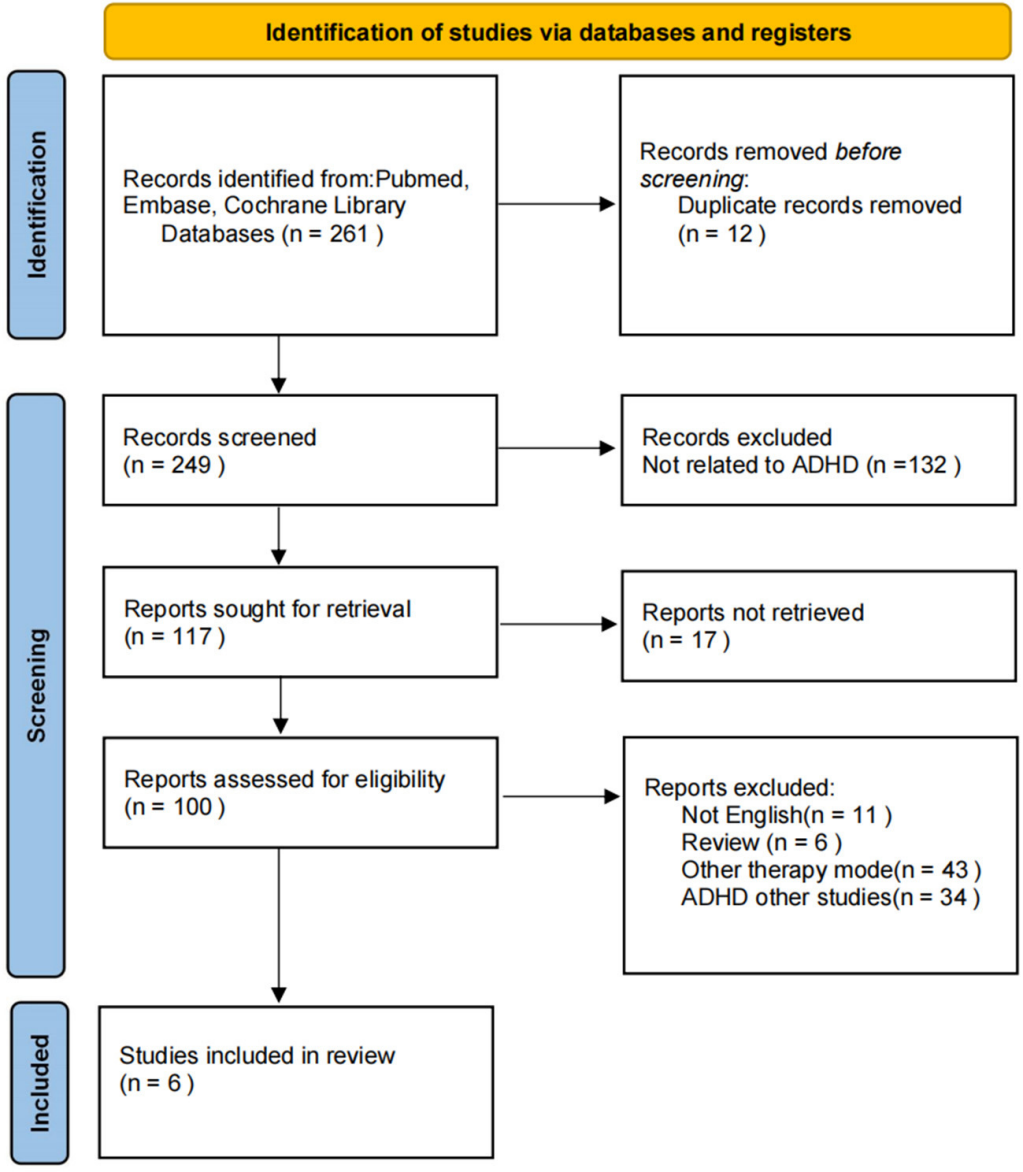
PRISMA flowchart of study identification, screening, assessment of eligibility and inclusion for synthesis.

Our search strategy searched 261 articles from the database, of which six studies met the review criteria (Figure 1). They carried out treatment programs through mobile phones, computers, and other electronic devices. One is designed as a game (McDermott et al., 2020), and two belong to behavioral cognitive therapy (Mo et al., 2015; Pettersson et al., 2017), two are parental education intervention (Dupaul et al., 2018; Franke et al., 2020), and one is teacher education intervention (Corkum et al., 2019). We list the author, sample size, intervention methods, and related characteristics in Table 1.

### The Effect Size of the Online Interbention

**Figure 2:**
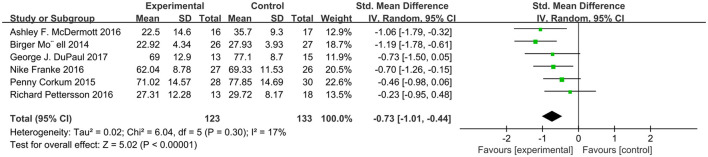
Attention score of online intervention ADHD compared with waiting list.

**Figure 3:**
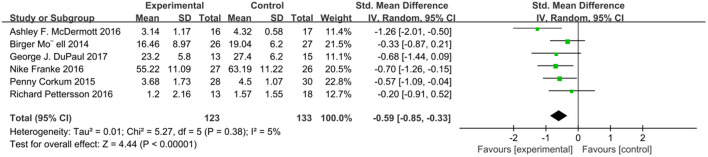
Social function score of online intervention ADHD compared with waiting list.

We analyzed ADHD scores in six studies using a random effects model (Figure 2), with a combined SMD of −0.73 (95% CI: −1.01, −0.44; *P* < 0.00001). The heterogeneity test *I*^2^ was 17%, (*P* = 0.30). In addition, we extracted relevant data from six studies and analyzed the subjects' social function related scores (Figure 3). The combined SMD was −0.59 (95% CI: −0.85, −0.33; *P* < 0.00001). The heterogeneity test *I*^2^ was 5%, (*P* = 0.38). Both of them belong to low heterogeneity.

### Subgroup Analysis

**Figure 4:**
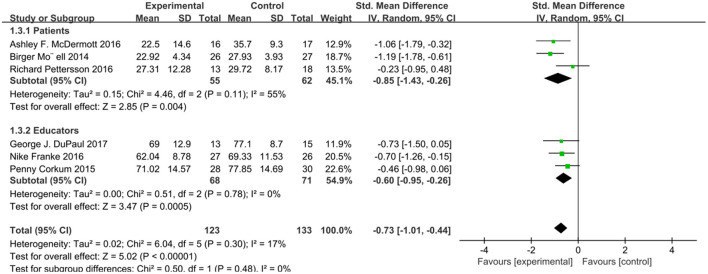
Subgroup analysis of attention score of online intervention ADHD compared with waiting list (for patients or educators).

**Figure 5:**
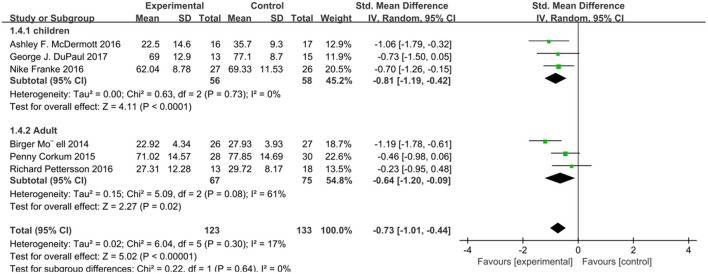
Subgroup analysis of attention score of online intervention ADHD compared with waiting list (for children or adults).

**Figure 6:**
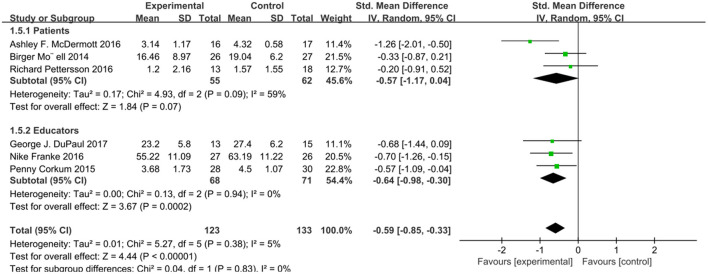
Subgroup analysis of social function score of online intervention ADHD compared with waiting list (for patients or educators).

**Figure 7:**
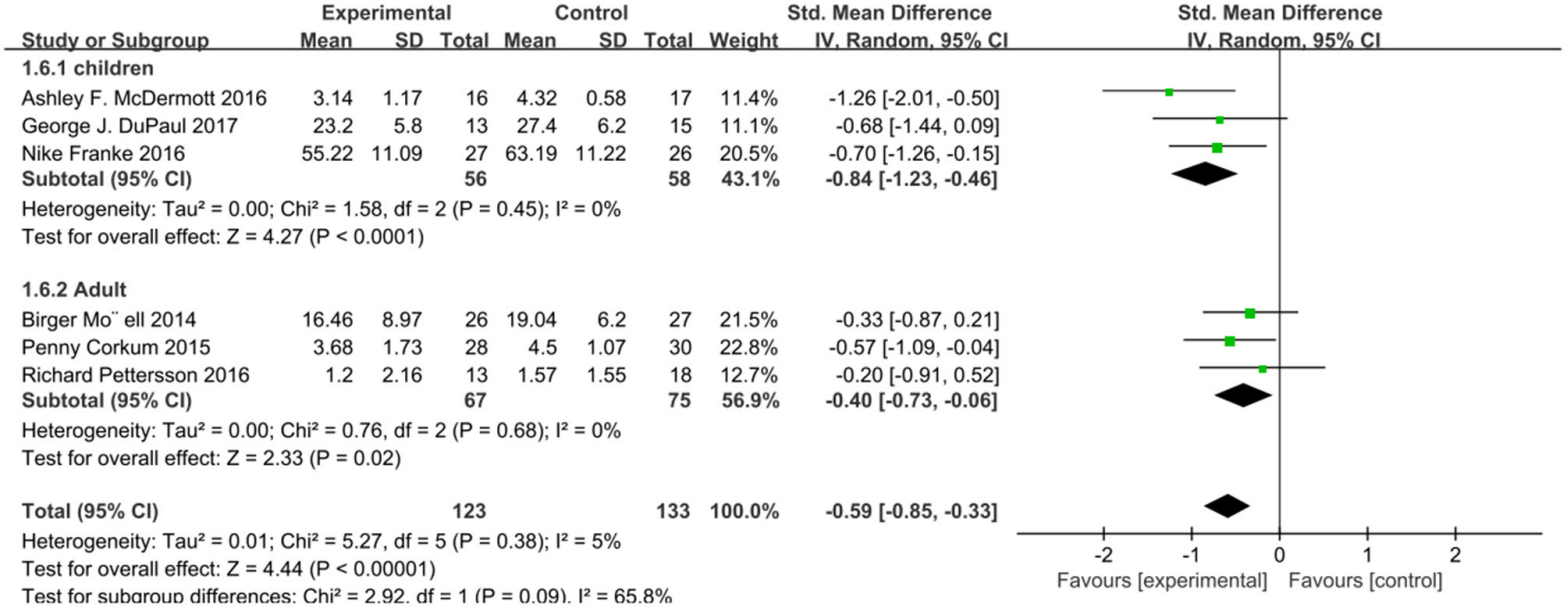
Subgroup analysis of social function score of online intervention ADHD compared with waiting list (for children or adults).

Considering the differences between adults and minors in age, psychology, physical development and other functions, we analyzed the subjects of different ages in groups. Considering that the intervention to the patients themselves in the network intervention belongs to direct intervention and the intervention to the patients' guardians belongs to indirect intervention, we conducted subgroup analysis on different intervention objectives. According to the intervention method, patient age, and test results, we conducted four subgroup analyses, and the scores of relevant scales were used as the outcome indicators. We analyzed four subgroups: attention score for patients and educators, *I*^2^ = 0% (*P* = 0.48), attention score for children and adults, *I*^2^ = 0 (*P* = 0.64), social function score for patients and educators, *I*^2^ = 0 (*P* = 0.83), and social function score for children and adults *I*^2^ = 65.8% (*P* = 0.09). There was no heterogeneity in these four subgroup analyses (Figures 4–7).

### Publication Bias

Egger's test in Stata 14 were used to analyze the publication bias of the study, and we drew the funnel plot (Supplementary Figures 3, 4). According to the egger's test of ADHD score, *P* = 0.851. According to the egger's test of the score of social function, *P* = 0.483. There was no publication bias in the study.

## Discussion

We searched a total of six studies that met the criteria and conducted a meta-analysis of six studies. We found that the response of ADHD patients after online intervention is better than that of the control group. This treatment can improve their attention and improve their social function, which may be a potential treatment.

At present, although some medical organizations in the United States (Pliszka, 2007), Canada (Edition, 2011), Latin America (Palacio et al., 2009), and Europe (Dalrymple et al., 2019) believe that drug treatment is very important, they still suggest that psychological and behavioral education intervention should be given priority in the treatment of patients. Although many drugs can be used to treat ADHD, statistics showed that 10–13% of patients had adverse reactions as early as the last century (Goldman et al., 1998), such as loss of appetite, insomnia, anxiety, irritability, or convulsions. For example, Atomoxetine may increase the risk of suicide, methylphenidate may hinder the growth and development of children, and clonidine may lead to hypotension (Daughton and Kratochvil, 2009). Under the epidemic of novel coronavirus pneumonia, the online intervention is particularly important (Kniffin et al., 2021). Especially behavioral therapy, educational support, psychological intervention, and other intervention methods are very suitable to be combined with the network for large-scale dissemination. In this study, we analyzed the efficacy of several online therapies through meta-analysis. We believe that the online intervention may be beneficial to current patients to reduce the symptoms of ADHD.

In addition to the test results, another objective of our study is to study the characteristics of effective interventions. In 3 of the 6 trials, educators for patients with ADHD showed the importance of the intervention environment. Since some of the participants included in the study are in school-age or even preschool stage, it is reasonable to have the influence of parents and teachers among the factors of intervention. Children of these ages are more likely to need some form of educator's help, including long-distance treatment and face-to-face intervention in life. A study has long shown that if children live in a conflict environment or abnormal parenting environment, it may lead to the symptoms of ADHD, and online intervention is more convenient to help children's families correct education and life problems.

The convenience of intervention implementation is the prominent advantage of online intervention. Before 2017, the number of global internet users had reached 3.5 billion. In front of users of this order of magnitude, even evidence-based medicine programs with low completion rates can provide considerable potential health impact (Rogers et al., 2018). But we found that there are few studies on the online treatment of ADHD in developing countries, which may be caused by the low network penetration and backward application equipment in developing countries (Carroll et al., 2017). However, with the development of online health programs and the progress of hardware facilities, developing countries may be closer and closer to telemedicine services, which will help to solve the problem of shortage and uneven distribution of medical resources, which will be very beneficial to developing countries (Hoque et al., 2020).

In addition to discussing the characteristics of each trial separately, we can try to integrate various online intervention to form a new comprehensive intervention. The targets of these interventions include the patient's parents, grandparents, and other guardians. Doctors can use the influence of family environment on ADHD children's attention and social function to reduce their negative emotions, improve their ability to pay attention to things in the process of growth, reduce the risk of children's future attention reduction, and provide a rehabilitation environment for attention deficit children. At the same time, doctors can help children's families create a good family atmosphere, let children learn appropriate skills and communication methods with their families in the process of daily communication, improve bad behavior problems, and make patients have enough physical and mental adaptability to adapt to social communication. At the same time, this kind of education may be aimed at educators such as teachers in the school or institution where the patient is located to create an appropriate learning atmosphere, which is not only effective for ADHD patients but also helpful for some patients having symptoms similar to ADHD, to help some patients with implicit attention deficit. The improvement of this way of education may not only help patients improve their attention problems, but also help patients improve their social skills, gradually adapt to the new social ways in the real society, improve and correct their related functions, improve people's tolerance and understanding of patients, and improve the humanistic quality of the whole society. In the intervention of the target population, we should not only carry out symptom trainings regularly but also formulate portable and feasible training procedures for the target population to help them train in their spare time at any time, improve their training initiative and promote their cooperation. In addition, we can also carry out psychological education for patients to help them understand the benefits of improving their ability, or the psychological factors affecting their attention, and help patients focus on improving their ability instead of focusing on various entertainment activities to consume their limited attention.

At present, genetic studies show that the pathogenesis of ADHD can not be explained by genetics (Posner et al., 2020). ADHD patients show heterogeneity in symptoms, some patients have impaired inhibitory function, and some patients have impaired working memory (Nigg et al., 2005). Considering that they have great differences in specific models, we can try to conduct case studies and adopt grounded theory or narrative medicine according to their life and growth experience, so as to explore more detailed pathogenic factors. Then we will conduct large-scale researches to help us find common characteristics from personal characteristics, to better understand the disease, develop targeted treatment plans and preventive measures, and the internet may be the best way to conduct such a research. Through subgroup analysis, we can see that the online intervention is equally effective for adult patients and children patients, which shows that the online intervention has a wide audience. We can also see that there may have differences in the intervention effects of adults and children on social functions, which may be caused by the different social roles of adults and children. We should give full play to the advantage of wide audience of online intervention and carry out targeted intervention content research and development for patients of different ages. For example, by adding games to the intervention, the fun of the game may attract more attention of attention deficit children and enable them to cooperate to complete the intervention. Adding skills training of different occupations to adult education can make patients of different occupations better practice the content of online intervention at work. In the future, with the development of online treatment technology, we can try to combine the diagnosis and investigation of ADHD with game modules, behavioral therapy, and other intervention means, combined with the education of patients' parents and teachers, and achieve all-round help by changing the way of patients and educators, so as to maximize the treatment effect. In the evaluation of attention function and social function of patients with tic disorder, the evaluation methods of various studies are different. In the future, more objective evaluation methods should be developed, and the same evaluation methods should be selected in clinical trials as far as possible, such as the same teacher evaluation, parent evaluation or patient self-evaluation. At the same time, the treatment of patients of different ages should be more targeted.

Our study has some limitations. In terms of evidence validity, due to the limited number of studies retrieved and the relatively small sample size, it may not be easy to fully explain the specific effect of ADHD online treatment and will affect the credibility of the results. In addition, although statistical studies do not show significant heterogeneity, there may also be heterogeneous, which is caused by factors such as race, gender, cultural background, and different statistical scales. The differences between some subjective factors and the scale may also affect the results, so these factors may lead to bias. Therefore, more researches are needed on the online treatment of ADHD. However, we believe that the development of online intervention is not without cost. In the research process, we rarely see statistical data describing the research and development cost of these studies. Doctors should comprehensively consider this factor to promote the popularization of online treatment technology.

## Conclusion

Through this study, we can see that the effects and methods of network treatment of ADHD are slightly different, but in general, it may be an effective treatment and has a lot of room for development. ADHD has its unique characteristics. It is a chronic disease, and the course of the disease can last until adulthood. Therefore, whether the treatment effect can be maintained at a certain level and whether there is corresponding continuous nursing is very important. The current research on the development of treatment is not enough, and the research on network interveners is not enough. At the same time, doctors may strengthen the case study of ADHD to help us better understand its internal mechanism.

## Data Availability Statement

The original contributions presented in the study are included in the article/Supplementary Material, further inquiries can be directed to the corresponding author/s.

## Author Contributions

SS: conception and design of the study. SS and YL: manuscript preparation and study conceptualization. SX: study design. NZ and JY: final revision and grammar editing. JD and JW: statistical analysis. All authors contributed to the article and approved the submitted version.

## Conflict of Interest

The authors declare that the research was conducted in the absence of any commercial or financial relationships that could be construed as a potential conflict of interest.

## Publisher's Note

All claims expressed in this article are solely those of the authors and do not necessarily represent those of their affiliated organizations, or those of the publisher, the editors and the reviewers. Any product that may be evaluated in this article, or claim that may be made by its manufacturer, is not guaranteed or endorsed by the publisher.

## References

[B1] Anastopoulos A. D. Dupaul G. J. Weyandt L. L. Morrissey-Kane E. Sommer J. L. Rhoads L. H. . (2016). Rates and patterns of comorbidity among first-year college students with ADHD. J. Clin. Child Adolesc. Psychol. 53, 1. 10.1080/15374416.2015.1105137 26852645 PMC4976041

[B2] Anastopoulos A. D. Sommer J. L. Schatz N. K. (2009). ADHD and family functioning. Curr. Atten. Disord. Rep. 1, 167. 10.1007/s12618-009-0023-2

[B3] Bidwell L. C. Mcclernon F. J. Kollins S. H. (2011). Cognitive enhancers for the treatment of ADHD. Pharmacol., Biochem. Behav. 99, 262–274. 10.1016/j.pbb.2011.05.002 21596055 PMC3353150

[B4] Carroll J. K. Moorhead A. Bond R. Leblanc W. G. Fiscella K. (2017). Who uses mobile phone health apps and does use matter? A secondary data analytics approach. J. Med. Int. Res. 19, e125. 10.2196/jmir.5604 28428170 PMC5415654

[B5] Corkum P. Elik N. Blotnicky-Gallant P. Mcgonnell M. Mcgrath P. (2019). Web-based intervention for teachers of elementary students with ADHD: randomized controlled trial. J. Atten. Disord. 23, 257–269. 10.1177/1087054715603198 26362259

[B6] Dalrymple R. A. Maxwell M. K. Russell S. Duthie J. (2019). NICE guideline review: Attention deficit hyperactivity disorder: diagnosis and management (NG87). Arch. Dis. Child. Educ. Pract. Ed. 105, 289–293. 10.1136/archdischild-2019-316928 31776172

[B7] Daughton J. M. Kratochvil C. J. (2009). Review of ADHD pharmacotherapies: advantages, disadvantages, and clinical pearls. J. Am. Acad. Child Adolesc. Psychiatry 48, 240–248. 10.1097/CHI.0b013e318197748f 19242289

[B8] Dupaul G. J. Kern L. Belk G. Custer B. Daffner M. Hatfield A. Peek D. (2018). Face-to-face versus online behavioral parent training for young children at risk for ADHD: treatment engagement and outcomes. J. Clin. Child Adolesc. Psychol. 47, S369-S383. 10.1080/15374416.2017.1342544 28715272

[B9] Dupaul G. J. Power T. J. Reid R. (1998). ADHD Rating scale—IV (for Children and Adolescents): Checklists, Norms, and Clinical Interpretation.

[B10] Edition T. (2011). Canadian ADHD Practice Guidelines.

[B11] Epstein J. N. Kelleher K. J. Baum R. Brinkman W. B. Peugh J. Gardner W. . (2016). Impact of a web-portal intervention on community ADHD care and outcomes. Pediatrics 138, e20154240. 10.1542/peds.2015-4240 27462065 PMC4960725

[B12] Epstein J. N. Langberg J. M. Lichtenstein P. K. Kolb R. Altaye M. Simon J. O. (2011). Use of an internet portal to improve community-based pediatric ADHD care: a cluster randomized trial. Pediatrics 128, e1201. 10.1542/peds.2011-0872 22007005 PMC3208964

[B13] Faraone S. V. Asherson P. Banaschewski T. Biederman J. Buitelaar J. K. Ramos-Quiroga J. A. . (2015). Attention-deficit/hyperactivity disorder. Nat. Rev. Dis. Primers 1, 15020. 10.1038/nrdp.2015.20 27189265

[B14] Franke N. Keown L. J. Sanders M. R. (2020). An RCT of an online parenting program for parents of preschool-aged children with ADHD symptoms. J. Atten. Disord. 24, 1716–1726. 10.1177/1087054716667598 27609783

[B15] Fuchs D. C. (1999). Attention-deficit hyperactivity disorder: a clinical workbook, J. Clin. Psychiatry 60, 127–128. 10.4088/JCP.v60n0211c

[B16] Goldman L. S. Genel M. Bezman R. J. Slanetz P. J. (1998). Diagnosis and treatment of attention-deficit/hyperactivity disorder in children and adolescents. Council on Scientific Affairs, American Medical Association. JAMA 279, 1100–1107. 10.1001/jama.279.14.1100 9546570

[B17] Higgins J. Green S. R. (2011). Cochrane Handbook for Systematic Review of InterventionsVersion 5.1.0.

[B18] Higgins J. P. T. Thompson S. G. Deeks J. J. Altman D. G. (2003). Measuring inconsistency in meta-analysis. BMJ 327, 557–560. 10.1136/bmj.327.7414.557 12958120 PMC192859

[B19] Hoque M. R. Rahman M. S. Nipa N. J. Hasan M. R. (2020). Mobile health interventions in developing countries: a systematic review. Health Informatics J. 26, 2792–2810. 10.1177/1460458220937102 32691659

[B20] Kessler R. C. Adler L. Ames M. Demler O. Faraone S. Hiripi E. . (2005). The World Health Organization Adult ADHD Self-Report Scale (ASRS): a short screening scale for use in the general population. Psychol. Med. 35, 245–256. 10.1017/S0033291704002892 15841682

[B21] Kessler R. C. Adler L. Barkley R. Biederman J. Conners C. K. Demler O. (2006). The prevalence and correlates of adult ADHD in the United States: results from the National Comorbidity Survey Replication. Am. J. Psychiatry 163, 716. 10.1176/ajp.2006.163.4.716 16585449 PMC2859678

[B22] Kniffin K. M. Narayanan J. Anseel F. Antonakis J. Ashford S. P. Bakker A. B. . (2021). COVID-19 and the workplace: Implications, issues, and insights for future research and action. The American psychologist, 76, 63–77. 10.1037/amp0000716 32772537

[B23] Kuja-Halkola R. Lind Juto K. Skoglund C. Rück C. Mataix-Cols D. Pérez-Vigil A. . (2021). Do borderline personality disorder and attention-deficit/hyperactivity disorder co-aggregate in families? A population-based study of 2 million Swedes. Mol. Psychiatry 26, 341–349. 10.1038/s41380-018-0248-5 30323291 PMC7815504

[B24] Matthys W. Cuperus J. M. van Engeland H. (1999). Deficient social problem-solving in boys with ODD/CD, with ADHD, and with both disorders. J. Am. Acad. Child Adolesc. Psychiatry 38, 311–321. 10.1097/00004583-199903000-00019 10087693

[B25] McDermott A. F. Rose M. Norris T. Gordon E. (2020). A novel feed-forward modeling system leads to sustained improvements in attention and academic performance. J. Atten. Disord. 24, 1443–1456. 10.1177/1087054715623044 26823382

[B26] Mo L. L. B Kollberg L. Nasri B. Lindefors N. Kaldo V. (2015). Living SMART — A randomized controlled trial of a guided online course teaching adults with ADHD or sub-clinical ADHD to use smartphones to structure their everyday life. Internet Interv. 2:1 (24–31). 10.1016/j.invent.2014.11.004

[B27] Molina B. Hinshaw S. P. Swanson J. M. Arnold L. E. (2009). The MTA at 8. J. Am. Acad. Child Adolesc. Psychiatry 48, 1123–1124. 10.1097/CHI.0b013e3181ba3e04 PMC3063150 19318991

[B28] Nigg J. T. Willcutt E. G. Doyle A. E. Sonuga-Barke E. J. (2005). Causal heterogeneity in attention-deficit/hyperactivity disorder: do we need neuropsychologically impaired subtypes? Biol. Psychiatry 57, 1224–1230. 10.1016/j.biopsych.2004.08.025 15949992

[B29] Page M. J. Mckenzie J. E. Bossuyt P. M. Boutron I. Hoffmann T. C. Mulrow C. D. . (2021). The PRISMA 2020 statement: an updated guideline for reporting systematic reviews. Int. J Surg. 88:105906. 10.31222/osf.io/v7gm2 33789826

[B30] Palacio J. D. Francisco D. Palacioscruz L. Ortizleón S. (2009). Algoritmo latinoamericano de tratamiento multimodal del trastorno por déficit de atención e hiperactividad (TDAH) a través de la vida. Rev. Colomb. Psiquiatr. 38(supl.1), 35–65. Available online at: http://www.scielo.org.co/scielo.php?script=sci_abstract&pid=S0034-74502009000500003&lng=pt&nrm=iso&tlng=es

[B31] Pettersson R. Söderström S. Edlund-Söderström K. Nilsson K. W. (2017). Internet-based cognitive behavioral therapy for adults with ADHD in outpatient psychiatric care. J. Atten. Disord. 21, 508–521. 10.1177/1087054714539998 24970720

[B32] Pliszka S. (2007). Practice parameter for the assessment and treatment of children and adolescents with attention-deficit/hyperactivity disorder. J. Am. Acad. Child Adolesc. Psychiatry 46, 894–921. 10.1097/chi.0b013e318054e724 17581453

[B33] Posner J. Polanczyk G. V. Sonuga-Barke E. (2020). Attention-deficit hyperactivity disorder. Lancet 395, 450–462. 10.1016/S0140-6736(19)33004-1 31982036 PMC7880081

[B34] Pringsheim T. Hirsch L. Gardner D. Gorman D. A. (2015). The pharmacological management of oppositional behaviour, conduct problems, and aggression in children and adolescents with attention-deficit hyperactivity disorder, oppositional defiant disorder, and conduct disorder: a systematic review and meta-analysis. Part 2: antipsychotics and traditional mood stabilizers. Can. J Psychiatry, 60, 52–61. 10.1177/070674371506000203 25886656 PMC4344947

[B35] Reimherr F. W. Roesler M. Marchant B. K. Gift T. E. Retz W. Philipp-Wiegmann F. . (2020). Types of adult attention-deficit/hyperactivity disorder: a replication analysis. J. Clin. Psychiatry 81, 19m13077. 10.4088/JCP.19m13077 32220152

[B36] Rogers M. A. Lemmen K. Kramer R. Mann J. Chopra V. (2018). Internet-delivered health interventions that work: systematic review of meta-analyses and evaluation of website availability. J. Med. Internet Res. 19, e90. 10.2196/jmir.7111 28341617 PMC5384996

[B37] Vander Stoep A. Myers K. (2013). Methodology for conducting the children's attention-deficit hyperactivity disorder telemental health treatment study in multiple underserved communities. Clin. Trials 10, 949–958. 10.1177/1740774513494880 23897950 PMC4286399

[B38] Weisman H. Qureshi I. A Leckman J. F Scahill L. (2013). Systematic review: Pharmacological treatment of tic disorders - Efficacy of antipsychotic and alpha-2 adrenergic agonist agents. Neurosci. Biobehav. Rev. 37, 1162–1171. 10.1016/j.neubiorev.2012.09.008 23099282 PMC3674207

[B39] Zima B. T. Bussing R. Tang L. Zhang L. Ettner S. Belin T. R. . (2010). Quality of Care for Childhood Attention-Deficit/Hyperactivity Disorder in a Managed Care Medicaid Program. J. Am. Acad. Child Adolesc. Psychiatry 49, 1225–1237.e11. 10.1016/j.jaac.2010.08.012 21093772 PMC3018146

