# Supplementary figures and images for: Efficacy of Online Intervention for ADHD: A Meta-Analysis and Systematic Review
Source: Front Psychol. 2022 Jun 28;13:854810. doi: 10.3389/fpsyg.2022.854810 (PMC9274127; doi:10.3389/fpsyg.2022.854810)

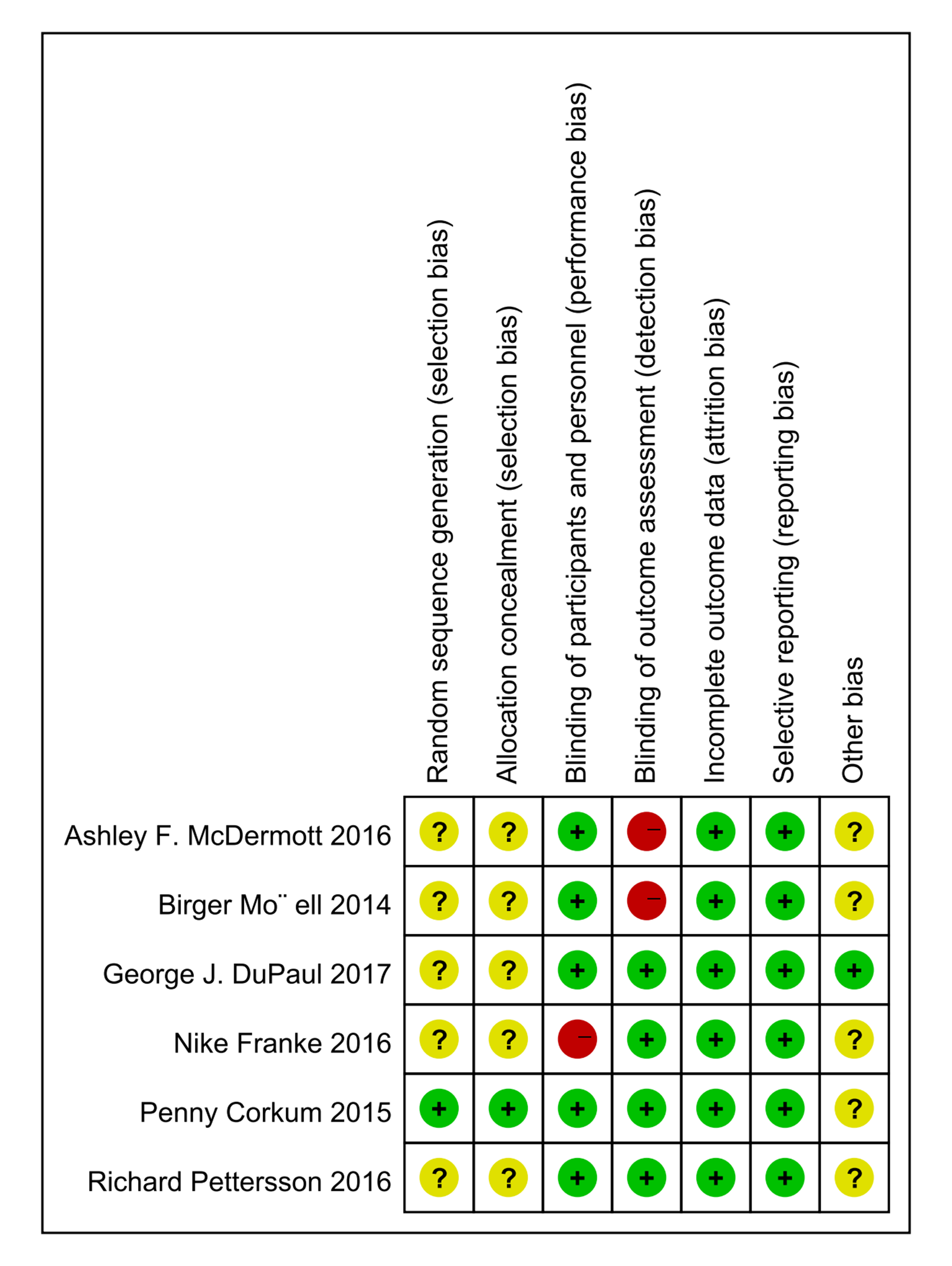

Supplement: Supplementary file 1 [file Figure_1.TIF]

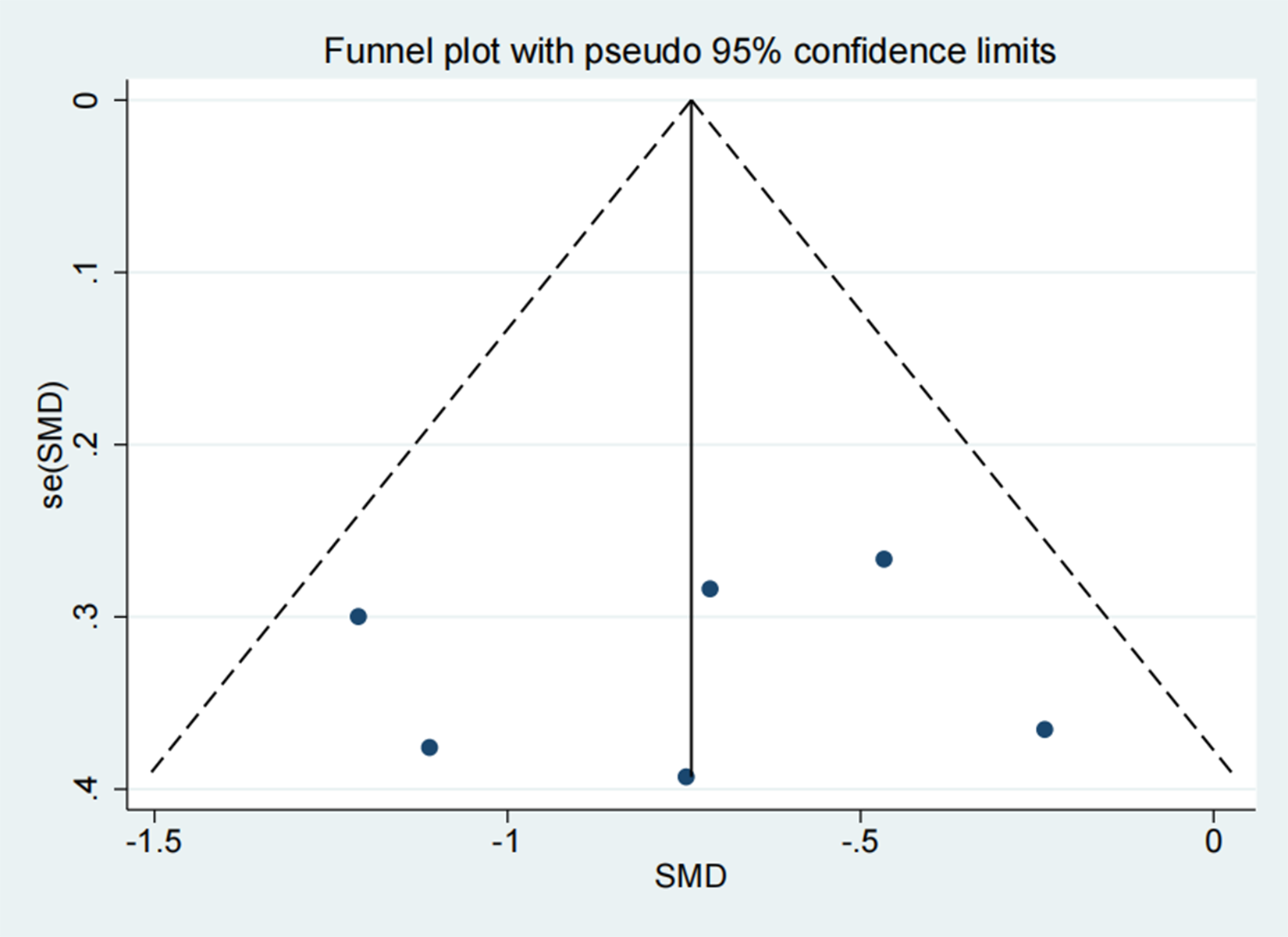

Supplement: Supplementary file 2 [file Figure_2.TIF]

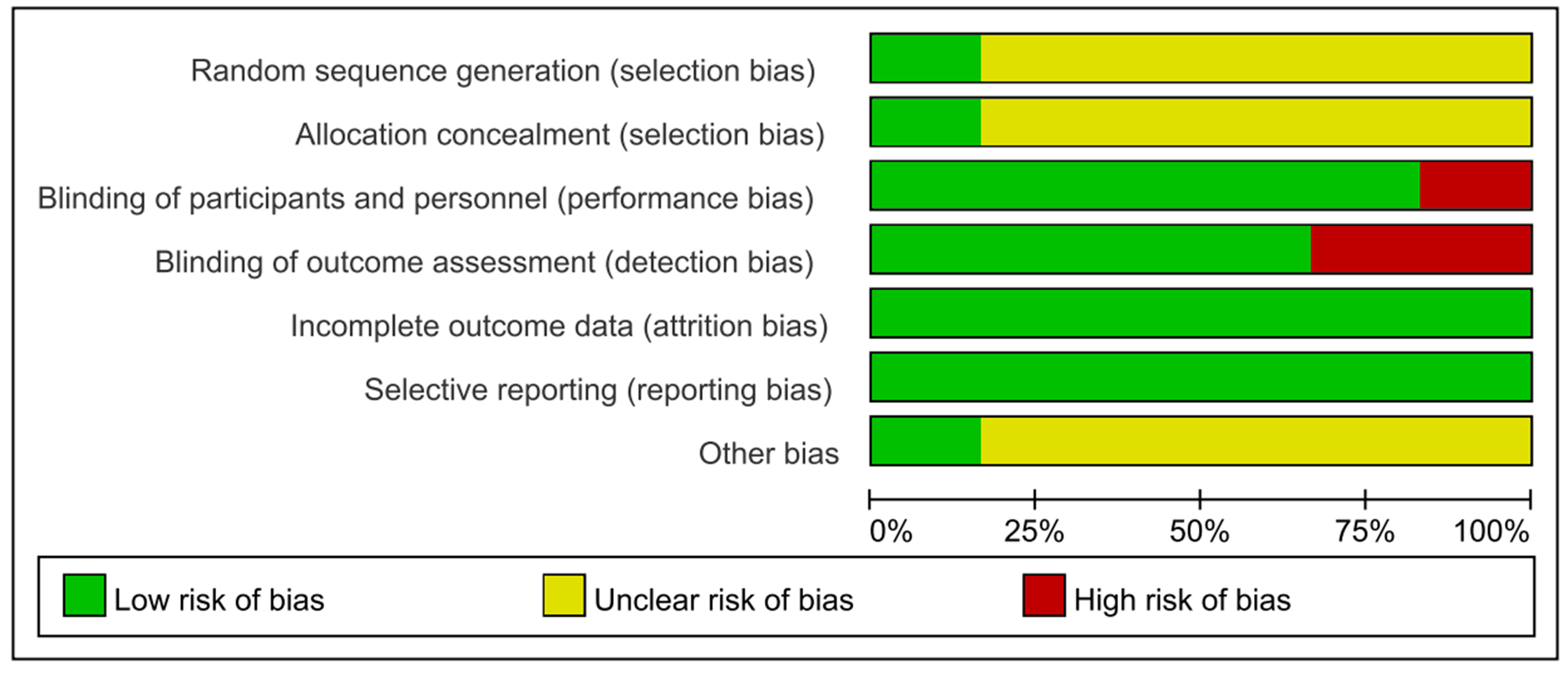

Supplement: Supplementary file 3 [file Figure_3.TIF]

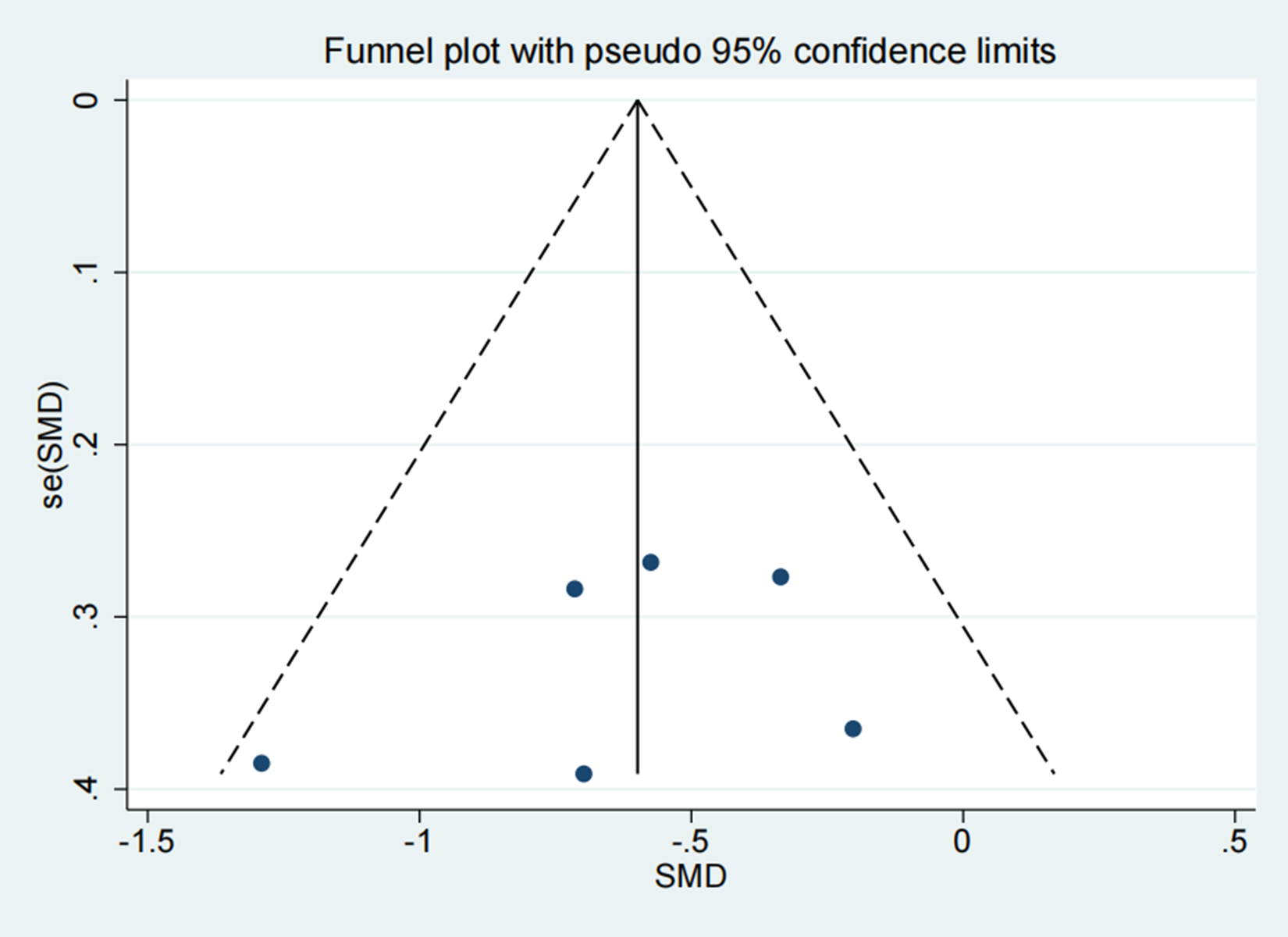

Supplement: Supplementary file 4 [file Figure_4.TIF]
